# Mechanistic Insights into the Wound Healing Activity of Plant Species in Diabetic Ulcers

**DOI:** 10.3390/cimb47120972

**Published:** 2025-11-24

**Authors:** Rodson Glauber Ribeiro Chaves, Fernanda Farias Costa, Letícia Andrade Fuchs, Lays Scherrer Rodrigues, Rhuan Antonio Nogueira Moraes, Paulo Sila da Silva Alves Junior, Márcia Cristina Goncalves Maciel, Flavia Maria Mendonça Amaral, Denise Fernandes Coutinho, Aramys Silva Reis

**Affiliations:** 1 Graduate Program in Health Sciences (PPGCS), Center for Biological and Health Sciences, Federal University of Maranhão, São Luís 65080-805, Maranhão, Brazil; rodson.glauber@ufma.br (R.G.R.C.);; 2 Nursing Department, State University of Maranhão, Balsas 65800-000, Maranhão, Brazil; 3 Graduate Program in Health and Technology (PPGST), Center for Biological and Health Sciences, Federal University of Maranhão, Imperatriz 65915-240, Maranhão, Brazil; 4 Faculty of Medicine, Center of Sciences of Imperatriz, Federal University of Maranhão, Imperatriz 65915-240, Maranhão, Brazil; leticia.fuchs@discente.ufma.br (L.A.F.); rhuan.antonio@discente.ufma.br (R.A.N.M.); paulo.sila@discente.ufma.br (P.S.d.S.A.J.)

**Keywords:** diabetic wound healing, plant extracts, molecular mechanisms, inflammation, angiogenesis, extracellular matrix remodeling

## Abstract

Diabetic foot ulcers represent a major complication driven by chronic inflammation, oxidative stress, impaired angiogenesis, and defective extracellular matrix remodeling. This integrative review synthesizes mechanistic evidence from 51 preclinical studies evaluating plant-derived interventions in diabetic animal models. Database searches (PubMed, Embase, Scopus) identified species modulating discrete molecular targets across healing phases. In the inflammatory phase, extracts suppressed NF-κB-dependent cytokine production (IL-1β, TNF-α, IL-6), reduced oxidative stress via Nrf2/HO-1 activation, and disrupted AGE-RAGE signaling, facilitating neutrophil resolution and macrophage reprogramming. During proliferation, interventions upregulated angiogenic factors (VEGF, bFGF, IGF-1) through ERK1/2 and PI3K/Akt-eNOS pathways, stimulated fibroblast proliferation, and enhanced collagen deposition. In the remodeling phase, extracts improved tensile strength by modulating MMP-2/9 and TIMP-2 balance, promoting type I collagen maturation and organized fiber architecture. Polyphenol-rich species (*Punica granatum*, *Quercus infectoria*, *Polygonatum kingianum*) demonstrated the most robust multi-target activity. However, translational gaps persist due to extract heterogeneity, limited phytochemical standardization, and overreliance on young male rodent models. Future development requires standardized bioactive quantification, dose–response characterization, advanced delivery systems (hydrogels, nanofibers), and validation in aged animals with comorbidities. These mechanistic insights support plant-based therapies as multi-component adjuvants targeting the complex pathophysiology of diabetic ulcers.

## 1. Introduction

Diabetic foot ulcers (DFU) are severe complications of diabetes driven by the convergence of peripheral neuropathy, vascular insufficiency, immune dysregulation, and impaired repair, leading to high rates of infection, hospitalization, and lower-limb amputation with substantial system costs [1,2]. Peripheral neuropathy, fueled by chronic hyperglycemia via oxidative stress and protein glycation, causes sensory, motor, and autonomic deficits that reduce pain perception, alter biomechanics, and impair sudomotor function, precipitating injury and delaying healing [3,4]. With diabetes affecting >10% of the population and current therapies proving inconsistent, there is an urgent need for new DFU treatment strategies [5].

Although various clinical practices are employed to prevent delays in the healing process, current therapeutic options remain limited, underscoring the urgent need for novel approaches. Given this scenario, interest in natural alternatives has grown exponentially, especially in plant-based products rich in bioactive compounds, such as flavonoids, alkaloids, terpenes, saponins, and tannins. These compounds act at various stages of the healing process through distinct mechanisms, exhibiting anti-inflammatory, antimicrobial, and antioxidant effects while promoting collagen synthesis, cell proliferation, and angiogenesis. The application of natural compounds via new systems can contribute to enhancements in wound treatment [6,7,8].

Numerous in vivo studies have demonstrated that plant-derived natural products can accelerate the healing process [9]. Experimental models play a critical role in validating these therapeutic agents. Chronic wound models in diabetic mice, for instance, simulate the pathophysiological and molecular aspects of human healing, thereby facilitating the evaluation of natural treatments in metabolically compromised contexts and providing essential data for research advancement [10].

Accordingly, this review evaluates the molecular mechanisms by which plant-derived interventions modulate oxidative stress, inflammatory cascades, angiogenesis, and extracellular matrix remodeling, synthesizes preclinical efficacy in diabetic animal models using standardized outcomes, and identifies links between mechanisms and efficacy to inform candidate selection and study design. Unlike prior overviews that address chronic wounds broadly, we focus on diabetic models and map phytochemical classes to discrete targets and healing readouts, outlining design recommendations that can accelerate natural products.

## 2. Materials and Methods

**Figure 1:**
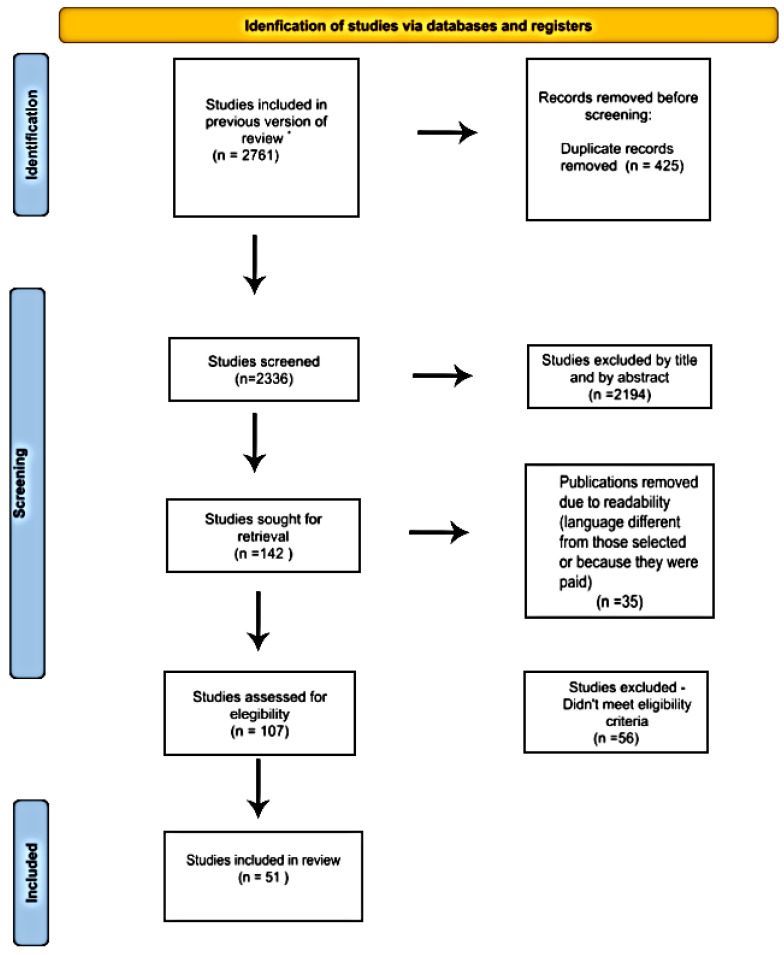
Study selection process PRISMA flowchart [11].

We conducted an integrative review between August 2023 and May 2025, to answer the central question: “In animal models of diabetic ulcers, how do plant extracts modulate the mechanisms of wound healing?”, structuring the work under the PCC framework—Population (plant species), Concept (wound healing), and Context (diabetic animal models). We searched PubMed, Embase, and Scopus using combinations of controlled descriptors and Boolean operators (“AND” and “OR”) without restrictions on publication year or language (Table 1, Figure 1).

**Figure 2:**
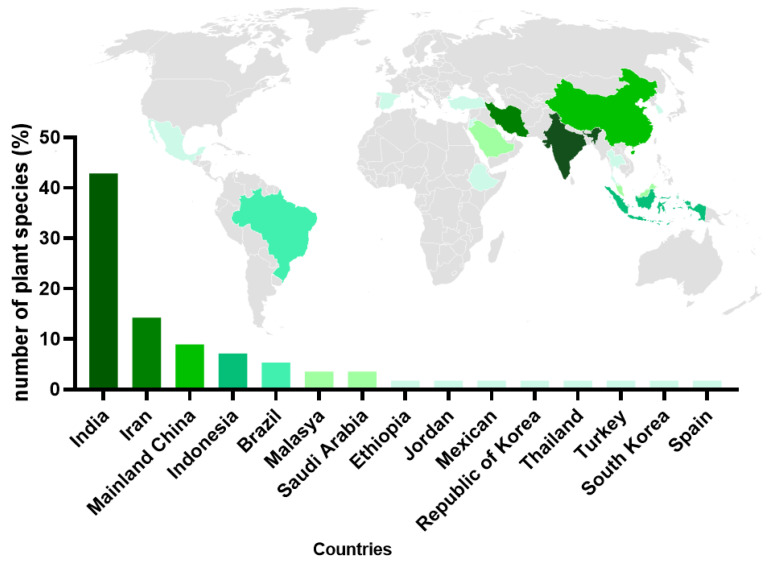
Global geographic distribution of articles on the effect of plant extracts on chronic wound healing. The various shades of green represent the number of articles published per country; they range from darker to lighter tones.

Records from all sources were managed in Rayyan QCRI with duplicate removal prior to screening. Two reviewers independently screened titles/abstracts and assessed full texts, with a third reviewer adjudicating disagreements. Studies were eligible if they reported primary in vivo experiments in diabetic animal models evaluating wound-healing after a plant-derived preparation (extract, fraction, or formulation), with full-text available in English or Portuguese and at least one wound-healing endpoint (e.g., wound area/closure, re-epithelialization, granulation tissue, collagen/hydroxyproline, tensile strength, angiogenesis, or inflammatory/oxidative biomarkers). We excluded studies without botanical identification, in vitro-only or non-diabetic/non-wound models, non-primary publications, duplicates, and articles without full text. Data were extracted using a standardized form (species/part, country, extract type/concentration, route, dose/duration, formulation, model/diabetes induction, outcomes, key findings, and citation) and tabulated in Microsoft Excel. Synthesis was qualitative, organized by mechanism (inflammation, angiogenesis, collagen/ECM remodeling, and oxidative stress) and intervention features (extract type and route). The search (including other sources) yielded 2761 records; 142 full texts were assessed, and 51 studies met eligibility (Figure 2). No protocol was prospectively registered. A formal animal-study risk-of-bias assessment was not performed.

As an integrative review, no protocol was prospectively registered. A formal animal-study risk-of-bias assessment was not undertaken due to substantial heterogeneity across models, interventions, and outcomes; instead, we narratively appraised key methodological elements.

## 3. Results

### 3.1. Results of Literature Search

The entire data collection process was conducted systematically, as presented in the following table (Table 2).

Among the included studies, 51 articles [12,13,14,15,16,17,18,19,20,21,22,23,24,25,26,27,28,29,30,31,32,33,34,35,36,37,38,39,40,41,42,43,44,45,46,47,48,49,50,51,52,53,54,55,56,57,58,59,60,61,62,63] had their data used for the preparation of the table, based on the extraction of the following information: plant species, part of the plant used, country of origin, type of extract, extraction method, formulation, concentration, route of administration, type of treatment, and treatment duration.

### 3.2. Origin of the Studied Plant Species

The geographical distribution of the plant species investigated reveals a clear regional concentration rather than a balanced global representation. Most studies originate from South and Southeast Asia, particularly India, followed by a smaller cluster from the Middle East, mainly Iran. Countries such as China, Indonesia, and Malaysia appear less frequently, while representation from Africa and the Americas is limited, with Brazil as the main contributor (Figure 2).

### 3.3. Plant Parts, Solvents, and Extraction Techniques

**Figure 3:**
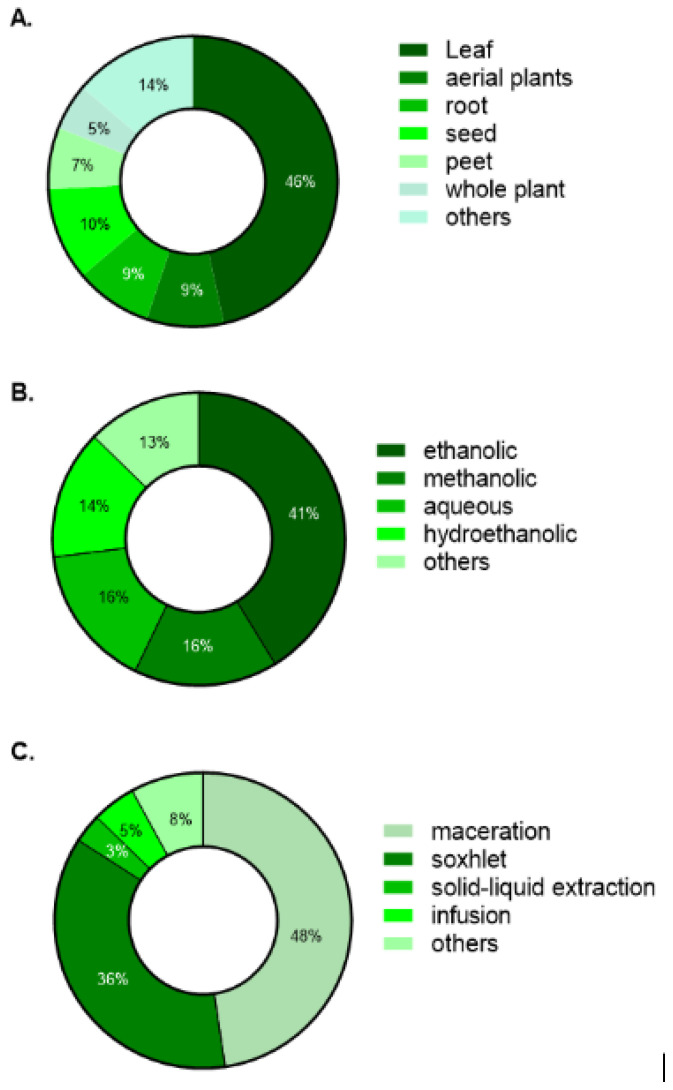
Characterization of: (**A**) the part of the plant species used; (**B**) the type of extract based on the solvents chosen; (**C**) the extraction method used in studies on the effect of plant extracts on chronic wound healing.

Leaves were the most frequently examined plant part, with other structures appearing less often (Figure 3A). Ethanolic and methanolic preparations predominate among solvents, with aqueous and hydroethanolic uses less common (Figure 3B). Maceration and Soxhlet extraction were the primary methods, while other procedures were used sporadically (Figure 3C).

### 3.4. Experimental Model

**Figure 4:**
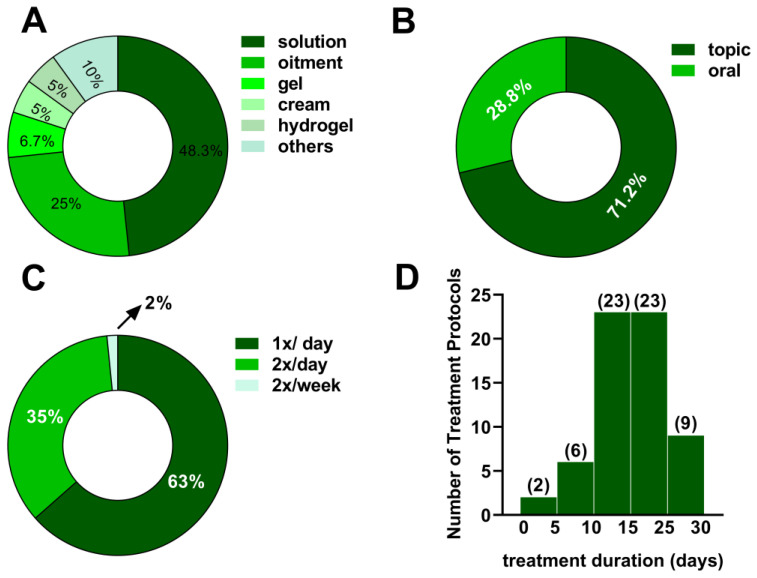
Charts showing: (**A**) formulation; (**B**) route of administration; (**C**) treatment frequency; (**D**) treatment duration related to the effect of plant extracts on chronic wound healing in in vivo hyperglycemia models.

Solutions and ointments were the most frequently used pharmaceutical forms for administering plant extracts (Figure 4A). The topical route predominated over oral administration (Figure 4B). Once-daily application was the most common treatment frequency (Figure 4C), and treatment durations typically ranged from ten to fifteen days (Figure 4D).

**Figure 5:**
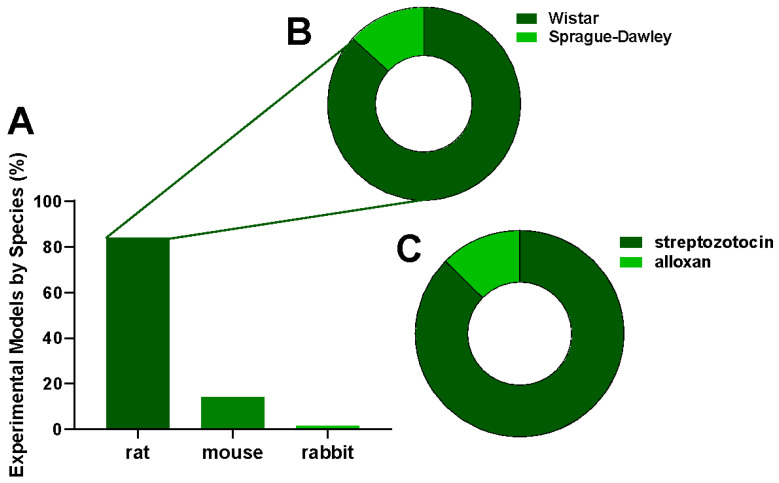
Characterization of (**A**) experimental model; (**B**) animal species; (**C**) hyperglycemia inducer related to the effect of plant extracts on chronic wound healing.

Rats were the most frequently used experimental model for diabetic wound healing, representing the majority of studies (Figure 5A). Among rat strains, Wistar was predominant (Figure 5B). Streptozotocin was the leading diabetogenic agent used to induce diabetes, while alloxan appeared less frequently (Figure 5C).

## 4. Discussion

### 4.1. Geographic and Methodological Overview

The mapping of plant species origins revealed a strong concentration in Asia, particularly in India, which accounted for about 43% of the species analyzed. This reflects the leadership of traditional Indian medicine and the cultural value attributed to medicinal plants [64]. In recent years, the development of Chinese herbal medicine has been challenged by shortages of resources and drug safety concerns related to end products. There have been significant efforts by Chinese scholars to tackle these challenges, which are revealed by analyzing the research trend of Chinese herbal medicine resources [65]. Brazil and other biodiverse regions, such as Latin America and Africa, have contributed a few species to the dataset. However, this distribution may also be influenced by publication bias and language indexing; many ethnobotanical studies from Africa and South America remain inaccessible or are published in local journals not indexed by the databases searched. Given the high prevalence of chronic diseases like diabetes in these regions, their native flora remains underexplored and warrants intensified investigation [66].

Most studies used leaf material, a choice considered more sustainable since leaves are renewable organs that can be harvested without destroying the plant, thereby ensuring species conservation and feasibility for repeated collections [67]. Ethanol and methanol were the predominant solvents, consistent with their ability to solubilize both polar and moderately non-polar compounds [68], enabling the recovery of a broader spectrum of phytochemicals relevant to wound healing. By contrast, aqueous extracts were less frequently tested in preclinical models, likely because water alone has limited efficiency in extracting lipophilic secondary metabolites [69]. Nevertheless, aqueous preparations such as decoctions and infusions remain central in traditional medicine, underscoring a gap between ethnopharmacological practice and experimental pharmacology that warrants further bridging [70].

Maceration and Soxhlet extraction were the most frequently employed techniques, largely due to their simplicity, reproducibility, and low cost, which make them particularly suitable for laboratories with limited infrastructure [69]. However, both methods have notable drawbacks, including long extraction times, high solvent consumption, and, in the case of Soxhlet, potential degradation of thermolabile compounds, limitations that underscore the need for more sustainable alternatives [71].

Topical formulations, including solutions, creams, gels, and ointments, were the most frequently employed, as they allow direct delivery of bioactive compounds to the wound site while minimizing systemic exposure [33,66]. In most studies, applications were performed once daily for 10–15 days, a period considered adequate to assess wound contraction and re-epithelialization in diabetic rodent models [72]. Oral preparations were also investigated for some species, particularly when systemic modulation of glycaemia or inflammation was anticipated [73].

Among experimental models, Wistar and Sprague-Dawley rats predominated, reflecting their reproducible inflammatory responses and well-established use in diabetes research [74]. Hyperglycaemia was most often induced with streptozotocin (STZ), which produces a robust model of type 1 diabetes. At the same time, some studies used alloxan or a combined STZ-nicotinamide protocol to approximate aspects of type 2 diabetes [75]. Taken together, these methodological choices enhance clinical relevance of the findings, while also highlighting the importance of developing phytotherapeutic strategies capable of addressing the multifactorial nature of diabetic wound healing.

### 4.2. Plant Species Modulating the Phases of Diabetic Wound Healing

Diabetic ulcers present an impaired healing response characterized by excessive production of reactive oxygen species (ROS), accumulation of advanced glycation end products (AGEs), and sustained activation of inflammatory pathways such as NF-κB [76]. Endothelial injury and capillary rarefaction limit tissue oxygenation, while fibroblasts exposed to high glucose generate a disorganized extracellular matrix. AGE crosslinking further stiffens tissue and disrupts integrin signalling, resulting in weaker scars [77].

In this review, we show that several plant species have emerged as promising modulators of these processes. Their bioactive compounds act through antioxidant, anti-inflammatory, angiogenic, and matrix-regulating mechanisms, directly addressing the main deficits observed in diabetic ulcers. Our analysis highlights that individual species often exert selective effects in distinct phases of healing.

By mapping these molecular actions across the different stages of repair, this review provides a framework to validate the therapeutic value of plant-derived interventions. Such an approach supports translational applications and guides the rational development of phytotherapeutic strategies tailored to the management of diabetic ulcers.

#### 4.2.1. Species Influencing the Inflammatory Phase

Diabetic wounds remain trapped in a hyper-inflammatory state because persistent hyperglycaemia stimulates keratinocyte IL-8 production via an EGFR–ERK pathway, increasing neutrophil recruitment and activation [78]. Post-prandial glucose spikes add further oxidative stress and trigger inflammatory gene expression through AGE and lipid-peroxidation-driven signalling [79]. AGEs interacting with their receptor RAGE keep macrophages in a pro-inflammatory phenotype and impair phagocytosis [80]. Consequently, controlling cytokine/ROS production, modulating leukocyte dynamics, and curbing AGE-RAGE signalling are key to shortening this phase.

**Modulation of Inflammatory Mediators and Oxidative/Nitrosative Stress**

Several extracts consistently attenuated early inflammatory and oxidative stress signals, but their effects vary in depth and evidentiary strength. *Quercus infectoria* gall ointments reduced plasma IL-6 and TNF-α and lowered wound malondialdehyde (MDA), while increasing total antioxidant capacity [54]. *Punica granatum* peel gel decreased wound NO and NOS activity (days 4–7) and improved antioxidant status [53], whereas *Phyllanthus emblica* cream diminished MDA and neutrophil infiltration with a trend to lower IL-6 [50].

Ethanolic or hydroethanolic extracts of *Polygonatum kingianum* not only attenuated inflammatory infiltrate but also suppressed AGEs and RAGE expression, downregulated multiple cytokines (TNF-α, IL-6, IL-2, IFN-γ) and activated the Nrf2/HO-1 antioxidant pathway [51]. Aqueous and ethanolic *Rosmarinus officinalis* extracts, whether given as essential oil or intraperitoneal infusion, curtailed early inflammatory changes and accelerated re-epithelialisation [56]. Hydroalcoholic ointments of *Onosma microcarpum* lowered inflammatory mediators and added antibacterial protection [48], while methanolic fractions of *Sida cordifolia* [57], hydroalcoholic leaf ointments of *Stachytarpheta jamaicensis* [58], and flavonoid-rich extracts of *Buddleja polystachya* [23] shortened the inflammatory window through combined antioxidant and antimicrobial effects. Oral extracts of *Teucrium polium* and *Aloe vera* reduced IL-1β and TNF-α and decreased MDA [15], and *Angelica dahurica* ethanolic extract lowered CD68^+^ macrophage infiltration and pro-inflammatory cytokines [17]. *Avena sativa* hydrogel scaffolds reduced inflammatory cell infiltration through avenanthramides and vitamin E [22], while *Cotinus coggygria* ointment decreased edema and inflammatory infiltrate and increased glutathione [28]. *Crocus pallasii* combined with topical methicillin decreased MDA and carbonyl proteins and enhanced SOD activity in infected wounds [29]. *Chrozophora tinctoria* topical and oral treatment reduced inflammatory infiltration and lowered glycaemia [27]. *Acalypha langinia* aqueous extract reduced congestion and edema in a dose-dependent manner [13]. These examples illustrate a common mechanism in which the modulation of oxidative and inflammatory mediators plays a central role. By reducing reactive oxygen and nitrogen species and dampening cytokine surges, the plant extracts foster a biochemical environment that limits neutrophil recruitment and favors the timely transition to the proliferative phase of healing.

**Regulation of Leukocyte Infiltration and Vascular Leakage**

Another group of plants acted primarily by resolving leukocyte persistence and microvascular disruption. *Stryphnodendron adstringens* crude bark gel (1% topical) up-regulated COX-2 early and increased VEGF by day 7, suggesting a controlled inflammatory burst that primes angiogenesis rather than perpetuating neutrophilia [60]. *Syzygium mundagam* 1–2% ointments reduced inflammatory cell counts by day 21 and shortened epithelialisation time (≈15.5 days at 2%) versus povidone-iodine [61]. Extracts of *Tridax procumbens* and *Typhonium trilobatum* promoted faster wound closure and reduced epithelialisation time [62,63]. In *T. trilobatum*, the methanolic and ethyl-acetate fractions exhibited superior healing activity compared with the chloroform fraction, indicating that the bioactive compounds responsible for tissue repair are likely concentrated in the more polar extracts. *Hydnocarpus wightiana* seed preparations reduced leukocytes and neutrophils at day 14 while stimulating macrophage cytokine release in vitro, indicating a transition from damaging neutrophilia to macrophage-mediated clearance [34]. *Ginkgo biloba* cream decreased inflammatory cell infiltration and prompted earlier scab thinning (day 9) [33]. *Garcinia mangostana* rind extract reduced TNF-α levels by 40–50% and slightly lowered glycaemia, further relieving neutrophil stimulation [32]. Oral *Centella asiatica* and *Ocimum sanctum* extracts combined glycaemic control with shorter epithelialisation [26,46]. Topically applied *Linum usitatissimum* oil reduced polymorphonuclear infiltration by day 14 [38], while *Lepidium meyenii* extracts, both oral and topical, decreased bacterial load and inflammatory cells [37], underscoring the importance of infection control for neutrophil resolution. *Jasminum grandiflorum* shortened closure time (day 14 vs. 20), implying mitigation of inflammatory delay [35]. In contrast, *Lantana camara* showed slower early contraction but eventually caught up, suggesting a modulated inflammatory timing rather than outright suppression [36]. These observations highlight that plants can modulate leukocyte dynamics through different pathways—early COX-2/VEGF activation, neutrophil suppression, macrophage reprogramming or improved microcirculation—and that timely resolution of congestion and edema is as important as cytokine suppression.

**Anti-AGE/RAGE Actions and Redox Balance**

Hyperglycaemia fosters AGE accumulation and RAGE signalling, which perpetuate inflammation and impair macrophage function. *Polygonatum kingianum* extracts stood out by simultaneously decreasing AGEs and RAGE and enhancing endogenous antioxidants via Nrf2/HO-1 [51]. *Olea europaea* ointments (2–5% Vaseline) increased total antioxidant capacity and accelerated scab fall, indicating that polyphenols (e.g., oleuropein) quenched ROS and broke AGE–RAGE loops [47]. Among *Onosma microcarpum* preparations, the n-hexane fraction showed a spectrum of neutrophil and microvascular responses: 30% extract retained high neutrophils at day 20 alongside abundant vessels, whereas 40% extract displayed fewer neutrophils with many microvessels, suggesting a regulated neutrophil-to-angiogenesis switch [48]. *Merremia mammosa* fractions, especially water extracts, improved early contraction and macrophage/fibroblast histology despite ongoing hyperglycaemia [41]; flavonoids likely conferred antioxidant protection. *Moringa oleifera* ointments reduced inflammatory mediators and offered antibacterial action [44], and *Ocimum sanctum* oral extract lowered glucose and provided antioxidant support [46]. These anti-AGE and redox-restoring effects target a fundamental driver of diabetic inflammation and illustrate that antioxidant capacity is not simply an add-on but a prerequisite for efficient inflammatory resolution.

**Antimicrobial Support**

Persistent infection sustains neutrophil infiltration and ROS generation [81]. *Onosma microcarpum* hydroalcoholic and acetone ointments inhibited bacterial growth while lowering inflammatory mediators [48]. *Merremia mammosa* water fractions reduced bacterial burden and improved macrophage/fibroblast balance [40,41], whereas *Lepidium meyenii* extracts (oral and topical) significantly lowered bacterial counts and inflammatory infiltration [37]. *Crocus pallasii* combined with methicillin further demonstrated that antimicrobial efficacy can synergise with antioxidant action to shorten inflammation [29]. These examples remind us that antimicrobial activity is not a secondary feature but integral to resolving the inflammatory bottleneck in diabetic wounds.

**Systemic Glycaemic Control and Metabolic Modulation**

Oral extracts such as *Teucrium polium*, *Aloe vera*, *Angelica dahurica*, *Ocimum sanctum*, *Centella asiatica*, *Moringa oleifera* and *Lepidium meyenii* improved fasting glucose or increased insulin levels, indirectly easing oxidative stress and cytokine production. *Acacia auriculiformis* methanolic hydrogel inhibited α-glucosidase and α-amylase [12]; by blunting post-prandial carbohydrate digestion, such inhibition can reduce post-prandial spikes that otherwise generate endothelial dysfunction and inflammatory reactions [82]. While the primary intent of these studies was wound healing, their systemic metabolic effects underscore the broader interplay between diabetes control and local inflammatory resolution.

**Other Species with Limited but Notable Inflammatory Data**

*Psoralea corylifolia* showed earlier wound-area reduction and a faster clinical course without biomarker reporting [52], suggesting anti-inflammatory potential. *Rehmannia glutinosa* alleviated carrageenan-induced inflammation and reduced ulcer area at day 8 in a diabetic foot model, indicating systemic anti-inflammatory activity [55]. *Caesalpinia bonducella* root extract promoted earlier inflammatory resolution, whereas bark and leaf extracts showed minimal benefit, highlighting the importance of the plant part and solvent [24]. *Cyclea peltata* methanolic extract improved early closure without measured biomarkers [24]. *Typhonium trilobatum*, *Tridax procumbens* and *Syzygium mundagam*—though lacking detailed molecular readouts—accelerated the clinical transition from inflammation to proliferation [61,62,63].

#### 4.2.2. Species Influencing the Proliferative Phase

In physiological repair, the proliferative phase begins once inflammation subsides. Fibroblasts migrate into the wound, proliferate, and deposit extracellular matrix (ECM), while keratinocytes migrate from the wound edges to resurface the defect. Myofibroblasts appear and drive wound contraction, and angiogenesis ensures oxygen and nutrient delivery. Hypoxia triggers hypoxia-inducible factor-1α (HIF-1α), inducing vascular endothelial growth factor (VEGF-A) and subsequent endothelial proliferation. In diabetes, however, reduced levels of IGF-1 and TGF-β impair fibroblast and keratinocyte recruitment, hyperglycaemia destabilizes HIF-1α and suppresses VEGF expression, macrophages are dysfunctional, and MMP/TIMP imbalance degrades ECM [83]. Consequently, granulation tissue is thin, angiogenesis is disordered, and re-epithelialisation is delayed. Against this backdrop, the plant extracts described below modulate key deficits—stimulating fibroblast proliferation and matrix deposition, promoting angiogenesis, and accelerating keratinocyte migration.

**Stimulation of Fibroblast Proliferation and Matrix Deposition**

Several extracts markedly enhanced fibroblast activity and collagen synthesis, counteracting the fibroblast dysfunction seen in diabetes. *Stryphnodendron adstringens* bark gel (1% topical) accelerated re-epithelialisation in Wistar rats, with complete coverage by days 10–14 and longer epithelial tongues at day 4, indicating rapid keratinocyte migration and active fibroplasia [60]. A separate hydroalcoholic extract (5%) of the same species delivered in hydrogel twice daily promoted angiogenesis and re-epithelialisation in diabetic and non-diabetic rats; in vitro, it stimulated fibroblast proliferation [59]. *Typhonium trilobatum* methanolic and ethyl-acetate fractions increased wound contraction and improved granulation histology relative to a chloroform fraction, demonstrating that polar constituents enhance fibroblast and keratinocyte responses [63]. *Tridax procumbens* ointment increased wound contraction and elevated hydroxyproline, total protein and DNA content, reflecting robust fibroblast activity and collagen biosynthesis that underpin granulation and re-epithelialisation [62]. *Syzygium mundagam* ointment increased wound contraction from day 10 onward and, histologically, boosted fibroblast proliferation and nascent capillaries [61]. *Stachytarpheta jamaicensis* hydroalcoholic ointment increased wound contraction, granulation mass and biosynthetic markers (hydroxyproline, hexosamine, total protein, DNA) [58]. *Sida cordifolia* hydrogel increased wound contraction and hydroxyproline while histology showed stronger epithelial growth and collagen deposition [57]. *Psoralea corylifolia* increased wound contraction and improved granulation and epithelial regrowth [52], whereas *Jasminum grandiflorum* oral extract boosted wound contraction, granulation tissue dry weight, neo-angiogenesis and hydroxyproline [35]. *Lantana camara* produced dose-dependent increases in contraction after day 7 and shortened epithelialisation, indicating support for keratinocyte migration and fibroplasia [36]. These examples show that many extracts act by restoring fibroblast proliferation and ECM production, addressing the impaired fibroblast function and collagen synthesis typical of diabetic wounds [83].

**Pro-angiogenic Signalling and Neovascular Support**

Angiogenesis is critically impaired in diabetic wounds due to suppressed HIF-1α and VEGF [83]. Several extracts countered this deficit by upregulating VEGF, basic fibroblast growth factor (bFGF) and other angiogenic signals. *Quercus infectoria* gall ointment increased VEGF mRNA, fibroblast density, angiogenesis, collagen deposition and rapid re-epithelialisation [54]. *Punica granatum* peel gel increased VEGF and EGF protein and mRNA (peaking around day 14), with early increases in hydroxyproline supporting collagen synthesis [53]. *Phyllanthus emblica* cream (10%) increased VEGF at day 7 and capillary density at days 7 and 14 [50]. *Rehmannia glutinosa* accelerated tissue regeneration with better scar formation and epithelialisation and increased capillary formation accompanied by elevated VEGF expression [55]. *Polygonatum kingianum* water and ethanolic extracts dramatically accelerated closure at days 3, 7 and 14, boosted angiogenic markers (CD34, VEGF, bFGF), thickened epidermis and dermis, and improved ECM turnover by decreasing MMP-2/9 and increasing TIMP-2 [51]. Phragmites vallatoria leaf ethanolic solution increased wet/dry granulation tissue mass and accelerated granulation, suggesting systemic pro-angiogenic support [49]. *Angelica dahurica* oral extract increased CD31^+^ vessel density and desmin^+^ pericyte recruitment in excisional wounds; mechanistic studies showed that the extract stimulated endothelial proliferation, migration and tube formation through ERK1/2 and PI3K/Akt-eNOS/NO signalling, without upregulating VEGF expression [17]. *Ginkgo biloba* cream achieved complete closure by day 13 (compared with 80% in controls) and histology showed a continuous epidermis with thicker regenerating dermis [33]. *Garcinia mangostana* produced ≈99% epithelial coverage by day 14, far exceeding diabetic controls; although direct angiogenesis markers were not reported, the accelerated closure implies enhanced neovascularisation and keratinocyte coverage [32]. *Nigella sativa* and *Moringa oleifera* ointments increased VEGF expression and reduced epithelialisation time [44,45], while *Mikania micrantha* nanogel accelerated closure in hyperglycaemic rats [42], and *Centella asiatica* oral extract promoted neovascularisation and granulation tissue formation [26]. *Avena sativa* scaffolds enhanced angiogenesis and epithelialisation via β-glucans and polyphenols [22], and *Buddleja polystachya* ointments increased angiogenesis and fibroblast activity [23]. *Centostigma macrophyllum* topical emulsion increased fibroblast numbers and blood vessels at day 7, with re-epithelialisation beginning by day 14 [25]. *Chrozophora tinctoria* increased wound contraction and granulation weights and histology showed greater angiogenesis [27]. *Cotinus coggygria* ointment accelerated granulation and re-epithelialisation and showed dilated neovessels [28]. *Crocus pallasii* methanolic ointment (often combined with methicillin in infected wounds) reduced wound area and improved fibroblast presence and neovascularisation [29]. *Linum usitatissimum* oil increased neovascularization scores by day 14 and earlier re-epithelialization [38], while *Lycium depressum* increased granulation quality and hydroxyproline by day 21 [39]. Together, these examples demonstrate that many extracts not only stimulate fibroblasts but also correct the diabetic deficit in angiogenesis.

**ECM Remodeling and Matrix Stabilisation**

Beyond generating granulation tissue, the quality of collagen and ECM is critical for long-term strength. *Punica granatum* gel increased hydroxyproline early, indicating robust collagen synthesis [53]. *Stachytarpheta jamaicensis* elevated hydroxyproline and hexosamine (ground substance) [58], *Sida cordifolia* increased hydroxyproline [57], and *Strephnodendron adstringens* hydroalcoholic gel improved collagen deposition [59]. *Onosma microcarpum* presented dose-dependent increases in fibroblasts and angiogenesis: n-hexane 30% showed the highest fibroblast density (≈1500/mm^2^), while n-hexane 40% had fewer neutrophils and more microvessels; acetone extract increased protein content and hydroxyproline; the ethanolic extract improved healing rate and collagen stabilisation; and the hydroalcoholic extract increased VEGF expression [48]. *Olea europaea* ointments increased wound contraction, granulation dry mass and protein content [47], while *Merremia mammosa* fractions improved angiogenesis and fibroblast density and accelerated matrix formation, with the water fraction outperforming the n-hexane and ethyl acetate fractions [40]. *Lepidium meyenii* topical ointment increased granulation tissue mass (wet/dry) and improved wound index, and its oral extract also increased granulation mass despite persistent hyperglycaemia [37]. *Hydnocarpi wightiana* extract, whether oral or topical, decreased wound area scores dose-dependently and improved granulation [34]. *Lantana camara* eventually achieved faster closure in the late proliferative window, despite slower early contraction [36]. *Cyclea peltata* methanolic extract and *Caesalpinia bonducella* root methanolic extract achieved ≈98–99% wound contraction by day 15 with marked epithelial closure, whereas bark and leaf ethyl-acetate extracts showed limited benefit [24], highlighting the importance of plant part and solvent. *Annona squamosa* ethanolic extract increased DNA, protein, collagen, hexosamine and uronic acid at the wound site, and histology showed early fibroblast and macrophage infiltration with accelerated epithelialisation [18]. *Aster koraiensis* oral extract accelerated closure and improved keratinocyte migration under hyperglycaemia [20], while *Astragalus fasciculifolius* gum cream increased healing ratio after day 14 [21]. *Acalypha langinia* aqueous extract reduced wound area and increased protein and DNA content in granulation tissue [13], and *Adhatoda vasica* and *Allium cepa* oral extracts enhanced closure, re-epithelialisation, tissue breaking strength, granulation weight and hydroxyproline [14].

#### 4.2.3. Species Influencing the Remodeling Phase

Diabetic wounds enter the remodeling phase burdened by deficits in collagen deposition and matrix maturation. Hyperglycaemia suppresses growth factors such as PDGF, leading to poor pruning and maturation of new capillaries, and diabetic scars exhibit lower collagen synthesis, altered fiber organization and reduced tensile strength. High matrix metalloproteinase activity and low TIMP levels further degrade the extracellular matrix, impeding replacement of type III collagen with type I and weakening scar integrity [84]. Plant extracts modulate these deficits via three principal mechanisms: strengthening tensile properties, enhancing collagen deposition and organization, and stabilizing matrix turnover.

**Strengthening Tensile Properties**

Several species improved biomechanical strength and cross-linking, directly addressing the reduced tensile integrity of diabetic scars [84]. *Typhonium trilobatum* methanolic and ethyl-acetate fractions yielded the highest breaking strength in diabetic incision models, outperforming chloroform fractions and implying superior collagen cross-linking and type I collagen replacement [63]. Topical *Tridax procumbens* increased tensile strength and maintained elevated hydroxyproline, total protein and DNA throughout closure, indicating sustained collagen synthesis and matrix consolidation [62]. *Sida cordifolia* hydrogel improved tensile strength and hydroxyproline in incision models, suggesting enhanced collagen maturation and cross-linking [57]. Oral and topical *Lepidium meyenii* increased hydroxyproline and hexosamine, with fibrocollagenous dermis and organized epidermis by day 21 [37], while topical *Lycium depressum* improved yield strength, ultimate strength, stiffness and maximum stored energy, confirming superior collagen maturation and scar stability [39]. *Acalypha langinia* increased incision tensile strength by 47–79% [13], and *Adhatoda vasica* and *Allium cepa* extracts elevated breaking strength and hydroxyproline in incision models, supporting robust collagen cross-linking [14]. *Psoralea corylifolia* and *Jasminum grandiflorum* significantly enhanced tensile strength and hydroxyproline levels, with histology showing an organized collagen architecture [35,52]. *Caesalpinia bonducella* root (methanol) and *Cyclea peltata* achieved near-complete closure with organized surface epithelium, suggesting improved matrix stabilization, whereas bark and leaf ethyl-acetate extracts showed limited gains [24]. Such biomechanical improvements are critical because diabetic scars typically have reduced tensile strength and poor contraction [84].

**Enhancement of Collagen Deposition and Organization**

Many extracts promoted organized collagen deposition and early scar maturation. *Stryphnodendron adstringens* increased mature type I collagen between days 10–14, yielding thicker, more organized fibers and improved dermal architecture despite hyperglycemia [60]. *Syzygium mundagam* achieved complete closure by day 21 with newly formed epidermis and organized collagen bundles, indicating early transition into remodeling [61]. *Rosmarinus officinalis* essential oil produced densely organized collagen bundles by day 15, whereas its aqueous extract improved collagen deposition and epidermal maturation but with less thick bundles, suggesting partial benefit [56]. *Quercus infectoria* improved collagen accumulation and epithelial coverage and upregulated VEGF mRNA and fibroblast density, which may promote matrix maturation [54]. *Punica granatum* peel gel achieved >90% closure by day 21, with histology showing re-epithelialization and neovascular maturation; combined with early increases in VEGF and hydroxyproline, these findings suggest strengthened early remodeling despite lacking tensile data [53]. *Rehmannia glutinosa* improved dermal thickness and organization, indicating enhanced early scar quality [55]. *Phyllanthus emblica* cream and *Phragmites vallatoria* solution accelerated closure and re-epithelialization, supporting earlier matrix reorganization [49,50]. *Polygonatum kingianum* water and ethanol extracts increased collagen density and improved dermal organization on Masson’s trichrome; they also shifted ECM turnover by downregulating MMP-2/9 and upregulating TIMP-2, suggesting stabilization of the matrix and improved scar quality [51]. *Olea europaea* ointments increased hydroxyproline and tissue transglutaminase, with correlations indicating that collagen deposition and cross-linking improved as glycaemic control and antioxidant status increased; these markers point to superior early remodeling [47]. *Onosma microcarpum* n-hexane preparations achieved near-complete closure by day 20, with organized dermis on histology; acetone and ethanolic fractions increased protein content and hydroxyproline, respectively, while hydroalcoholic extract upregulated VEGF, together suggesting multi-faceted support for collagen deposition despite absent tensile data [48]. *Merremia mammosa* topical fractions increased collagen fiber density and sustained closure up to day 25; the water fraction gave the most complete closure by day 11, implying rapid entry into remodeling [40,41]. *Ocimum sanctum* increased tissue strength (“strength of the fabric”), implying better collagen cross-linking; its systemic effects complicate attribution but suggest sustained matrix maturation [46]. *Linum usitatissimum* oil produced organized collagen and nearly complete closure by day 14 [38], and *Lantana camara* achieved complete closure by day 15, implying earlier onset of remodeling though tensile metrics were lacking [36].

**Matrix Turnover and MMP/TIMP Modulation**

Excessive matrix metalloproteinases and insufficient tissue inhibitors hamper diabetic remodeling [84]. *Polygonatum kingianum* ethanolic extract reduced MMP-2/9 and increased TIMP-2, while water extract lowered AGEs, RAGE and inflammatory cytokines; these shifts favour stable collagen turnover and durable matrix organization [51]. *Annona squamosa* ethanol extract decreased MMP-2 and MMP-9 expression both in vivo and in keratinocyte culture, restoring dermal thickness and organization [18]. *Aster koraiensis* oral extract reduced MMP-2/9 expression and activity, leading to improved structural organization and inferred tensile strength [20]. *Astragalus fasciculifolius* gum increased the healing ratio and exhibited improved epithelialization and collagen deposition, though tensile data were inferred rather than measured [21]. *Avena sativa* scaffolds promoted collagen deposition and fiber organization in diabetic wounds, suggesting balanced matrix synthesis and degradation [22].

**Early Transition into Remodeling and Dermal Organization**

Many species accelerated closure and re-epithelialization, allowing the wound to enter the remodeling phase sooner. *Merremia mammosa* water fraction produced near-complete closure by day 11 [41], *Ginkgo biloba* achieved 100% closure by day 13 with organized collagen and dermis [33], and *Garcinia mangostana* showed ≈99% epithelial coverage by day 14 [32]. *Centella asiatica* improved granulation breaking strength and hydroxyproline with organized collagen bundles [26]. *Hydnocarpus wightiana* treatment resulted in no visible ulcers by two weeks, implying earlier remodeling, but lacking mechanical data [34]. *Buddleja polystachya* increased tensile strength in the incision model and accelerated wound closure [23], while *Centostigma macrophyllum* wounds exhibited a compact dermis and collagen fiber deposition by day 28 [25]. *Chrozophora tinctoria* increased hydroxyproline and tensile strength with organized fibers [27], *Cotinus coggygria* improved hydroxyproline and dermal organization [28], and *Crocus pallasii* improved hydroxyproline and tensile properties with organized collagen and higher epithelialization scores [29]. Additional species such as *Angelica dahurica* [17], *Aloe vera* (nanofibrous dressings) [16], *Aster koraiensis* [20], *Acalypha auriculiformis* [12], *Avena sativa* [22], *Annona squamosa* [18], *Buddleja polystachya* [23], *Centostigma macrophyllum* [25], *Chrozophora tinctoria* [27], *Cotinus coggygria* [28], and *Crocus pallasii* [29] also promoted collagen deposition, dermal reorganization, and tensile strength through various extracts and formulations.

### 4.3. Recommendations for Translational Development

The plant extracts summarized here act on multiple targets across these phases, suppressing inflammatory cytokines and oxidative stress, promoting fibroblast proliferation and angiogenesis, and enhancing collagen deposition and crosslinking. A critical limitation of the current evidence base is the marked heterogeneity across studies along with methodological inconsistencies and the absence of standardized wound models and endpoints. These factors complicate cross-study comparisons and inflate apparent effect sizes, underscoring the need for harmonized preclinical standards. To translate these findings into clinically useful therapies, three overarching considerations are critical. In parallel, explicit recognition of translational barriers should guide study design from bench to bedside.

First, natural products should be developed as multi-component and multi-target adjuvants rather than single-agent cures. Many of the studied extracts (e.g., *Quercus infectoria* [54], *Polygonatum kingianum* [51], *Onosma microcarpum* [48]) exhibit combined antioxidant, anti-inflammatory, pro-angiogenic and matrix-modulating properties, suggesting that their real value may be in complementing existing care. Traditional polyherbal formulations already exploit such synergy: a recent polyherbal gel containing *Ficus racemosa*, *Emblica officinalis*, *Curcuma longa*, *Carica papaya*, *Terminalia bellerica*, *Acacia catechu* and *Aloe vera* healed 85% of diabetic rat wounds by day 16 and outperformed povidone-iodine [85]. Combining plant extracts with advanced delivery systems such as hydrogels, nanofibers, or nanoparticles can further enhance efficacy and reduce toxicity. For example, silver or gold nanoparticles conjugated to phytochemicals have shown synergistic antimicrobial and prohealing effects [85]. Future translational research should therefore focus on optimized combinations that address inflammation, infection, angiogenesis and matrix deficits simultaneously, and should evaluate synergy with existing treatments (e.g., growth factors, offloading devices, debridement). Mechanistic validation should accompany these combinations, employing targeted signaling assays and multi-omics to confirm on-target activity and identify predictive biomarkers.

Second, rigorous standardisation and quality control of extracts are essential. Efficacy varied markedly with plant part, solvent and dose; for instance, *Caesalpinia bonducella* root extract outperformed bark and leaf extracts [24], and *Onosma microcarpum* n-hexane and hydroalcoholic fractions produced distinct histological and vascular responses [48]. Many studies lacked phytochemical characterisation, making it impossible to correlate outcomes with active constituents. Translational development demands batch-to-batch consistency with defined marker compounds, sustainable harvesting, and scalable extraction methods. Dose-finding studies are needed to identify therapeutic windows and avoid paradoxical effects (e.g., high neutrophil counts with *Onosma* n-hexane 30% vs. 40% [48]). Moreover, formulations should be designed for clinical practicality—for example, ointments, creams and nanofibers should use biocompatible excipients and have defined pharmacokinetics. Where feasible, in vitro–in vivo correlation (IVIVC) and pharmacokinetic/pharmacodynamic (PK/PD) modeling should be incorporated to bridge preclinical dosing to human exposure.

Third, preclinical models must better mirror human diabetic wounds. The literature indicates that most studies have employed young male Wistar or Sprague–Dawley rats with streptozotocin-induced diabetes, a model that primarily heals through contraction and lacks chronicity. This narrow focus partly explains why many promising therapies have not advanced beyond murine models [86]. New translational research should incorporate aged animals, comorbidities (such as obesity, infection, neuropathy, and ischemia), and models, such as splinted wounds in pigs or rabbits, that heal by granulation. In vitro platforms using human diabetic fibroblasts, keratinocytes and macrophages, or bioengineered skin equivalents, could provide mechanistic insight and support dose optimisation. Standardised endpoints should include not only wound area and hydroxyproline but also tensile strength, type I/III collagen ratio, MMP/TIMP balance, growth factor expression and long-term scar function. Adoption of shared core outcome sets and blinded histopathology, alongside preregistered protocols, would increase reproducibility and predictive validity. The bench-to-bedside gap will only close when preclinical outcomes are designed to predict human efficacy.

Finally, clinical translation requires interdisciplinary collaboration. Natural product researchers, wound-care clinicians, pharmacologists and regulatory scientists must work together to design early-phase trials that integrate phytochemicals with standard care and advanced wound dressings. Studies should be randomised, blinded and adequately powered, and should monitor systemic effects (e.g., glycaemia, liver and renal function) to ensure safety. Regulatory pathways may be smoother if extracts are positioned as adjuncts or nutraceuticals rather than drugs. Given the high unmet need and the multifactorial pathogenesis of diabetic wounds, plant-based therapies that have demonstrated multi-phase efficacy provide an attractive avenue—but only if developed with scientific rigor and translational foresight. In sum, addressing heterogeneity and methodological inconsistency, explicitly tackling animal-to-human barriers, and coupling efficacy claims to mechanistic validation and clinical trials are the key steps to credible translation.

## 5. Conclusions

This review provides a comprehensive mechanistic overview of plant species with wound-healing activity in diabetic ulcers. The evidence indicates that several plant species show the strongest mechanistic evidence of benefit. These species modulate key molecular targets by suppressing NF–κB-dependent inflammation, activating antioxidant pathways (Nrf2/HO1), stimulating angiogenic factors (VEGF, bFGF), and regulating extracellular matrix remodeling through a balance of MMPs and TIMPs, as well as collagen cross-linking.

However, the current evidence remains limited by heterogeneity in extract preparation, dosage, animal models, and reporting of outcomes. Future studies should prioritize the standardization of extract composition and dose, the quantification of bioactive compounds, and the inclusion of mechanistic endpoints, such as cytokine profiles, oxidative stress biomarkers, and collagen organization indices. Establishing comparative dose–response curves and conducting cross-species validation would further strengthen the translational potential. Integrating phytochemical standardization with modern experimental designs—such as controlled diabetic wound models, omics-based pathway analysis, and biocompatible delivery systems (e.g., hydrogels or nanofibers), will be essential to move from descriptive evidence toward clinically viable phototherapeutic strategies.

## Figures and Tables

**Table 1:** Search Strategies.

Database	Search Strategies
PUBMED	((“chronic wound” [MeSH Terms] OR (“chronic wound healing” OR ((“diabetic” [MeSH Terms] OR (“diabetic wound healing”) ((“extract plant” [MeSH Terms] OR (“natural product OR (“plant extract”) OR (“human” [mesh terms] AND (“in vivo” OR in vitro model))
EMBASE	(chronic wound OR “chronic wound healing” OR diabetic wound healing) AND (“extract plant” OR natural product of plant extract)
SCOPUS	(chronic wound) AND (chronic wound healing AND “diabetic wound healing” OR (extract plant OR “natural product” OR plant extract)

**Table 2:** Distribution of the selected data according to plant species, plant part, country of origin, extract type, extraction method, formulation, concentration, route of administration, treatment duration, findings, and citation.

Plant Species	Part	Country-Origin	Extract Type	Extraction Method	Formulation/Concentration	Route of Administration	Treatment/Time (Days)	Finds	Reference
*Acacia* *auriculiformis* A. Cunn. ex Benth.	Leaf	India	Methanolic	Maceration	Hydrogel (10%)	Topical	1x/day (15)	↓ activity of α- glucosidase and α-amylase ↑ rate of lesion contraction—dose-dependent ↓ time of epithelialization ↓ levels of hydroxyproline	[12]
*Acalypha langinia* Müll.Arg.	Leaf	México	Aqueous	Soxhlet	Solution (0.05–0.5%)	Oral	2x/day (7)	↓ congestion and edema ↓ wound area—dose-dependent ↑ protein and DNA content in granulation tissue ↑ tensile strength of incision wounds	[13]
*Adhatoda nees*	Leaf	India	Alcoholic	n.i. *	Solution (400 mg/kg)	Oral	1x/day (11)	↑ wound closure ↑ re-epithelialization ↑ tensile strength ↑ granulation tissue weight ↑ hydroxyproline	[14]
*Allium cepa* (L.)	Bulb	India	Alcoholic	n.i. *	Solution (300 mg/kg)	Oral	1x/day (11)	↑ wound closure (day 11) ↑ re-epithelialization ↑ tensile strength ↑ granulation tissue weight ↑ hydroxyproline	
*Teucrium* (L.)	Aerial parts	Iran	Hydro- ethanolic	Soxhlet	Ointment (5%–10%)	Topical	1x/day (14)	↓ IL-1β, TNF-α ↓ MDA ↑ fibroblasts/collagen ↑ VEGF, IGF-1, FGF-2, GLUT-1 ↑ tensile strength	[15]
*Aloe vera* (L.) Burm.f.	Leaf	Iran	Hydro- ethanolic	Soxhlet	Ointment (5%–10%)	Topical	1x/day (14)	↓ IL-1β, TNF-α ↑ VEGF, IGF-1, GLUT-1 ↑ fibroblasts/collagen ↑ wound closure ↑ tensile strength	
*Aloe vera* (L.) Burm.f.	Leaf	Spain	Hydro- ethanolic	Maceration	Nanofiber (5–10%)	Topical	2x/day (8)	↑ Fibroblast proliferation ↑ Reepithelisation ↑ Wound closure ↑ Resolution of chronic inflammation Mild ↑ collagen deposition	[16]
*Angelicae* *dahuricae*	Root	China	Ethanolic	Percolation	Solution (20%)	Oral	1x/day (14)	↓healing time ↓ CD68^+^ macrophages; ↓ IL-1β/TNF-α ↑ Granulation and re-epithelialization ↑ Angiogenesis (↑ CD31 vessels; ↑ pericyte recruitment) ↑ HUVEC proliferation/migration/tube formation (ex vivo/in vitro) ↑ Collagen I deposition	[17]
*Annona squamosa* (L.)	Seed	India	Ethanolic	Maceration	Solution (n.i. *)	Oral	1x/day (14)	↑ DNA, protein, collagen, hexosamine, uronic acid ↑ Fibroblasts, macrophages, angiogenesis ↑ Wound contraction and epithelialisation ↑ Tensile strength and collagen organization ↓ Lipid peroxidation	[18]
*Anthocephalus* *cadamba*	Leaf	India	Aqueous	Infusion	Solution (500 mg/kg)	Topical	1x/day (28)	↓area of injury ↑ contraction rate ↑ regeneration, neovascularization, collagen deposition and fibroblast proliferation	[19]
*Aster koraiensis*	Aerial parts	Republic of Korea	Ethanolic	Maceration	Solution (100 mg/kg)	Oral	1x/day (18)	↑ Wound closure (day 14) ↑ Keratinocyte migration ↑ Skin thickness/organization ↓ MMP-2/9 expression and activity ↑ Re-epithelialization	[20]
*Astragalus* *fasciculifolius*	Gum	Iran	Aqueous	Infusion	Cream (5–10%)	Topical	1x/day (20)	↑ Wound healing ratio (day 14–20) ↑ Granulation tissue formation ↑ Epithelialization ↑ Collagen deposition/organization ↑ Tissue restoration	[21]
*Avena sativa* (L.)	Seed	India	Ethanolic	Maceration	Hydrogel (200 mg/kg)	Topical	2x/day (14)	↓ Inflammatory infiltration and oxidative stress ↑ Fibroblast adhesion and proliferation ↑ Wound contraction ↑ Angiogenesis and epithelialization ↑ Collagen deposition and fiber organization	[22]
*Buddleja* *polystachya* (F.)	Leaf	Ethiopia	Methanolic	Maceration	Ointment (5–10%)	Topical	1x/day (18)	↓ Inflammatory exudate and scab duration ↑ Wound contraction ↑ Fibroblast activity and angiogenesis ↑ Epithelialization rate ↑ Tensile strength and collagen organization	[23]
*Caesalpinia* *bonducella* (L.) Fleming	Root	India	Methanolic	Soxhlet	Solution (50–100 mg/kg)	Topical	1x/day (15)	↓ blood glucose ↑ contraction of the injury ↑ healing	[24]
	Bark	India	Ethylacetate	Soxhlet	Solution (51–100 mg/kg)	Topical	1x/day (15)	↓ blood glucose ↑ contraction of the injury	
	Leaf	India	Ethylacetate	Maceration	Hydrogel (52–100 mg/kg)	Topical	1x/day (15)	↓ blood glucose ↑ contraction of the injury	
*Cyclea peltata* (L.)	Leaf	India	Methanolic	Soxhlet	Solution (50 mg/kg)	Topical	1x/day (15)	↓ inflammatory period ↑ wound contraction ↑ granulation tissue formation ↑ epithelial closure	
*Cenostigma macrophyllum* Tul.	Leaf	Brazil	Hexanic	Solid–liquid extraction	Emulsion (0.5%)	Topical	1x/day (28)	↓ inflammatory cells/resolution of infiltrate ↑ nitric oxide ↑ fibroblasts and granulation tissue ↑ angiogenesis and onset of re-epithelializatio ↑ wound size reduction	[25]
*Centella asiatica* (L.) Urb.	Leaf	India	Ethanolic	Soxhlet	Solution (200 mg/kg)	Oral	2x/day (14)	↓ fasting blood glucose ↑ wound contraction; ↓ epithelialization time ↑ granulation tissue (wet/dry weight) ↑ breaking strength (granulation) ↑ hydroxyproline and collagen organization	[26]
*Chrozophora* *tinctoria* (L.)	Leaf	India	Hydro- methanolic	Soxhlet	Solution (5%)	Oral	1x/day (21)	↓ inflammatory infiltration; ↓ inflammatory period ↑ wound contraction; ↓ time to epithelialization ↑ granulation tissue (wet/dry weight) and total protein ↑ collagen (hydroxyproline) ↑ tensile strength	[27]
*Cotinuos coggygria* (S.)	Leaf	Turkey	Ethanolic	Soxhlet	Solution (200 mg/kg)	Oral	2x/day (14)	↓ inflammatory infiltrate/edema ↑ GSH ↓ MDA ↑ re-epithelialization and angiogenesis ↑ hydroxyproline	[28]
*Crocus pallasii* (S.)	Leaf	Iran	Methanolic	Maceration	Ointment (2%)	Topical	2x/day (14)	↓ MRSA CFU/g ↑ Wound contraction ↓ wound area ↑ Fibroblasts and neovascularization ↑ Hydroxyproline ↑ Biomechanics	[29]
*Cynodon* *dactylon* (L.) Pers.	Whole plant	India	n.i. *	Maceration	Ghrita (40%)	Topical	1x/day (21)	↑ contraction of the injury ↓ epithelialization time	[30]
*Dodoneae viscosa* (J.)	Leaf	India	Ethanolic	Maceration	Ointment (10%)	Topical	2x/day (16)	↑ rate of contraction of the injury ↑ collagen content ↑ anti-inflammatory activity	[31]
*Garcinia mangostana* (L.)	Peel	Indonesia	Ethanolic	Maceration	Solution (25%)	Oral	1x/day (14)	↓ TNF-α; controlled inflammatory rise ↓ fasting blood glucose ↑ wound closure (≈99% at day 14 vs. ≈64% control) ↑ re-epithelialization speed (inferred from coverage) → remodeling onset earlier; no tensile/collagen data	[32]
*Ginkgo biloba* (L.)	Leaf	China	Aqueous	Soxhlet	Cream (1–5%)	Topical	1x/day (13)	↓ inflammatory cells/scab duration ↑ wound contraction (100% by day 13) ↑ re-epithelialization (higher histology score) ↑ collagen alignment/organization ↑ overall healing rate vs. control	[33]
*Hydnocarpus* *wightiana* Blume	Seed	South Korea	Hydro- ethanolic	Soxhlet	Solution (50 mg/kg)	Topical	1x/day (14)	↓ WBC/neutrophils (day 14) ↑ macrophage IL-12/TNF-α (in vitro) ↓ wound area score; dose–response → benefit independent of glycemia → earlier remodeling (closure ≤ 2 weeks)	[34]
*Jasminum* *grandiforum* (L.)	Flower	India	Ethanolic	Maceration	Solution (250 mg/kg)	Topical	1x/day (11)	↑ wound contraction (days 7–14) ↑ granulation dry weight ↑ hydroxyproline (collagen) ↑ incision breaking strength ↑ neo-angiogenesis (histology)	[35]
*Lantana camara* (L.)	Leaf	India	Ethanolic	Soxhlet	Ointment (10%, 15% and 20%)	Topical	1x/day (23)	→ early contraction lag (week 1) ↑ wound contraction after day 7 (dose-dependent) ↓ epithelialization time ↑ complete closure by day 15–17 (higher doses) → remodeling inference; no tensile/collagen data	[36]
*Lepidium* (L.)	Root	India	Hydro- ethanolic	Maceration	Solution (200 mg/kg)	Oral	1x/day (30)	↓ bacterial load (days 7 & 14) ↓ inflammatory cell infiltration ↑ wound contraction and wound index ↑ granulation tissue (wet/dry weights) ↑ hydroxyproline & hexosamine (collagen/ECM)	[37]
	Root		Hydro- ethanolic	Soxhlet	Ointment (5–10%)	Topical	2x/day (30)	↓ bacterial load (days 7 & 14) ↓ inflammatory cell infiltration ↑ wound contraction (from day 10; dose-dependent) ↑ granulation tissue (wet/dry weights) ↑ hydroxyproline & hexosamine (collagen/ECM)	
*Linum usitatissimum* (L.)	Seed	Malasya	Aqueous	Infusion	Oil (200 mg/kg)	Topical	2x/day (14)	↓ inflammatory cell infiltration (day 14) ↑ re-epithelialization (early; day 4 diabetic) ↑ surface closure rate ↑ neovascularization (day 14) ↑ collagen organization (histology)	[38]
*Lycium* (L.)	Leaf	Iran	Methanolic	Maceration	Ointment (500 mg/kg)	Topical	2x/day (14)	↑ antioxidant activity ↓ area of injury ↑ collagen deposition ↑ epithelialization and vascularization ↑ cell proliferation ↑ acute hemorrhage and edema scores	[39]
*Merremia macrocarpa* (L.) Roberty.	Tuber	Indonesia	Aqueous	Maceration	Solution (0,05%)	Topical	1x/day (21)	↑ wound healing % (day 10; 50–100 mg ≈ positive control) ↑ angiogenesis ↑ fibroblast density ↑ collagen fiber density (ECM) ↓ inflammatory delay (dose-responsive)	[40]
*Merremia macrocalyx* (Ruiz & Pav.) O’Donell.	Leaf	Indonesia	Ethanolic	Ultrasonic-assisted	Solution (10%)	Topical	1x/day (5)	↑ wound healing % (water fraction 93.4% at day 11) ↓ wound size (days 7–11 vs. control) ↑ contraction/epithelialization speed (inferred) ↑ water fraction > n-hexane > ethyl acetate (day 11) ↑ performance ≈ gentamicin at day 11	[41]
*Mikania* *micrantha* Kunth.	Leaf	Indonesia	Ethanolic	Maceration	Nanogel (2%)	Topical	1x/day (1)	↑ healing rate	[42]
*Mimosa pudica* (L.)	Leaf	India	Ethanolic	Maceration	Solution (200 mg/kg)	Oral	2x/day (7)	↓ inflammatory mediators ↑ antibacterial activity ↓ wound area ↓ time to epithelialization ↑ VEGF	[43]
*Moringa oleifera* Lam.	Leaf	Malasya	Ethanolic	Maceration	Ointment (0.5%, 1% and 2%)	Topical	1x/day (21)	↑ antibacterial activity ↓ area of injury dose-dependent ↓ epithelization time ↓ levels of inflammatory mediators ↑ VEGF expression	[44]
*Nigella* (L.)	Seed	Iran	Ethanolic	Maceration	Ointment (20–40%)	Topical	1x/day (14)	↓ wound area ↓ healing time ↑ epidermal thickness	[45]
*Ocimum* (L.)	Leaf	India	Ethanolic	Soxhlet	Solution (800 mg/kg)	Oral	1x/day (7)	↓ wound area ↓ time to epithelialization ↑ granulation tissue weight ↑ hydroxyproline ↑ tensile strength	[46]
*Olea europaea* (L.)	Leaf	Saudi Arabia	Ethanolic	Maceration	Ointment (2–5%)	Topical	2x/day (21)	↓ epithelialization time ↑ wound contraction ↑ granulation tissue (dry weight, protein) ↑ hydroxyproline and tTG (collagen deposition/cross-linking) ↑ TAC; closure/scar positively correlated with HPX/tTG/TAC ↑antioxidant Capacity	[47]
*Onosma* *microcarpum* (D.)	Root	Iran	Hexanic	Soxhlet	Ointment (20%, 30%, 40% and 60%)	Topical	1x/day (20)	↑ wound closure (day 20) ↑ fibroblasts (up to ~1500/mm^2^) ↑ angiogenesis (up to ~200 vessels/mm^2^) ↓ residual wound area vs. base → remodeling improvement inferred (no tensile/HPX)	[48]
	Root		Acetone	Soxhlet	Ointment (30%)	Topical	1x/day (20)	↓ area of injury ↓ time to epithelialization ↑ protein content (granulation) ↑ hydroxyproline (collagen) ↑ fabric strength	
	Root		Ethanolic	Maceration	Ointment (30%)	Topical	1x/day (20)	↑ healing rate ↓ time to epithelialization ↑ collagen stability ↑ antioxidant capacity → remodeling quality improved (surrogate)	
	Root		Hydro- ethanolic	Maceration	Ointment (30%)	Topical	1x/day (20)	↑ antibacterial activity ↓ area of injury (dose-dependent) ↓ time to epithelialization ↓ inflammatory mediators ↑ VEGF expression	
*Phragmites* Adans.	Leaf	India	Ethanolic	Soxhlet	Solution (400 mg/kg)	Oral	2x/day (11)	↑ granulation tissue (wet/dry) ↓ time to epithelialization ↑ wound contraction ↓ wound area	[49]
*Phyllanthus* (L.)	Fruit	Thailand	Ethanolic	Maceration	Cream (10%)	Topical	1x/day (30)	↓ MDA ↓ neutrophils ↑ VEGF ↑ capillary vascularity ↑ wound closure/re-epithelialization	[50]
*Polygonatum* *kingianum*	Rhizo me	China	Aqueous	Soxhlet	Gel (2–8 g/kg)	Topical	1x/day (28)	↓ AGEs/RAGE; ↓ TNF-α, IL-6, IL-2, IFN-γ ↑ Nrf2/HO-1, SOD, GSH, T-AOC; ↓ MDA ↑ wound closure (days 3, 7, 14) ↑ CD34/VEGF/bFGF, angiogenesis; ↑ epidermis/dermis thickness ↓ MMP-2/9; ↑ TIMP-2; ↑ collagen density	[51]
	Rhizo me		Ethanolic	Soxhlet	Gel (2–8 g/kg)	Topical	1x/day (28)	↓ AGEs/RAGE; ↓ TNF-α, IL-6, IL-2, IFN-γ ↑ antioxidant status (Nrf2/HO-1; SOD/GSH/T-AOC); ↓ MDA ↑ wound closure (days 3, 7, 14) ↑ CD34/VEGF/bFGF; ↑ epidermis/dermis thickness ↓ MMP-2/9; ↑ TIMP-2; ↑ collagen density	
*Psoralea* (L.)	Whole plant	India	Ethanolic	Maceration	Ointment (1%)	Topical	1x/day (9)	↑ wound contraction ↑ granulation and epithelial regrowth ↑ tensile strength ↑ collagen organization ↓ overall healing time vs. control	[52]
*Punica granatum* (L.)	Peel	Saudi Arabia	Methanolic	Maceration	Gel (5%)	Topical	2x/day (21)	↓ NO/NOS; ↑ antioxidant status ↑ VEGF/EGF (protein & mRNA) ↑ hydroxyproline (early) ↑ wound contraction/closure (>90% by day 21) ↑ re-epithelialization and vascular maturation	[53]
*Quercus* (L.)	Galls	Iran	Hydro- ethanolic	Maceration	Ointment (5–10%)	Topical	1x/day (14)	↓ IL-6/TNF-α; ↓ MDA; ↑ TAC ↑ VEGF; ↑ fibroblasts; ↑ angiogenesis ↑ collagen deposition ↑ re-epithelialization → improved early matrix organization	[54]
*Rehmannia* *glutinosa* (L.)	Root	China	Aqueous	Decoction	Solution (n.i. *)	Oral	2x/day (30)	↓ inflammation (carrageenan model) ↓ ulcer area (day 8) ↑ VEGF and apillaries ↑ epithelialization/scar quality ↑ dermal organization (early remodeling)	[55]
*Rosmarinus officinalis* (L.)	Aerial parts	Jordan	Aqueous	Hydrodistillation	Essential oil (5–10%)	Topical	2x/day (3)	↓ inflammation/faster re-epithelialization ↑ granulation tissue ↑ angiogenesis ↑ wound contraction ↑ collagen organization	[56]
	Aerial parts		Aqueous	Solid–liquid extraction	Solution (10%)	Topical	1x/day (3)	↓ inflammation/faster re-epithelialization ↑ granulation tissue ↑ wound contraction ↑ collagen deposition ↓ blood glucose (systemic)	
*Sida cordifolia* (L.)	Aerial parts	India	Methanolic	Maceration	Hydrogel (10%)	Topical	1x/day (20)	↓ time to epithelialization ↑ wound contraction ↑ hydroxyproline (collagen) ↑ tensile strength ↑ epithelial/collagen histology	[57]
*Stachytarpheta* *jamaicensis* (L.) Vahl	Leaf	India	Hydro-ethanolic	Soxhlet	Solution (2–5%)	Topical	2x/day (20)	↓ time to epithelialization ↑ wound contraction ↑ granulation tissue mass ↑ collagen/hexosamine/protein/DNA ↑ tensile strength	[58]
*Stryphnodendron* *adstringens* (Mart.) Coville	Peel	Brazil	Hydro- ethanolic	Maceration	Gel (5%)	Topical	2x/day (16)	↑ angiogenesis ↑ re-epithelialization ↑ fibroblast proliferation ↑ overall healing progression	[59]
*Stryphnodendron* *adstringens* (Mart.) Coville	Leaf	Brazil	Ethanolic	Maceration	Solution (1%)	Oral	2x/day (14)	↑ COX-2 (d4–10) and VEGF (d7) ↑ keratinocyte migration/proliferation; complete re-epithelialization by d10–14 ↑ type I collagen and fiber organization dermal permeation confirmed (topical gel) overall earlier inflammatory–proliferative transition	[60]
*Syzygium aqueum* (Burm.f.) Alston	Peel	India	Methanolic	Soxhlet	Ointment (1–2%)	Topical	1x/day (21)	↓ inflammatory persistence; ↓ epithelialization time (~15.5 d at 2%) ↑ wound contraction (from d10) ↑ fibroblasts and neovascularization (histology) ↑ complete closure by d21 ↑ collagen bundle organization	[61]
*Tridax procumbens* (L.)	Leaf	India	Ethanolic	Soxhlet	Solution (2.5–5%)	Topical	2x/day (14)	↓ wound index; ↓ epithelialization time ↑ wound contraction ↑ hydroxyproline/protein/DNA (granulation) ↑ tensile strength (incision model) ↑ overall healing rate (diabetic)	[62]
*Typhonium* *trilobatum* (L.)	Whole plant	India	Methanolic	Soxhlet	Solution (100 mg/kg)	Topical	1x/day (9)	↓ epithelialization time (diabetic; MeOH/EtOAc > CHCl3) ↑ wound contraction (MeOH/EtOAc) ↑ granulation/epithelial coverage (histology) ↑ tensile strength (incision; MeOH/EtOAc > CHCl3) effective under infected diabetic wounds (excision)	[63]

↑ sign means increased. ↓ sign means decreased. * n.i.: not informed. Taxonomic: http://floradobrasil.jbrj.gov.br/. URL ( accessed on 12 November 2025).

## Data Availability

The original contributions presented in this study are included in the article. Further inquiries can be directed to the corresponding authors.

## Abbreviations

The following abbreviations are used in this manuscript:

AGEs	Advanced Glycation End Products
bFGF	basic Fibroblast Growth Factor
CAPES	Coordination for the Improvement of Higher Education Personnel
CAT	Catalase
CD31	Cluster of Differentiation 31
CD34	Cluster of Differentiation 34
CD68	Cluster of Differentiation 68
CFU/g	Colony Forming Units per gram
CNPq	Brazilian National Council for Scientific and Technological Development
COX-2	Cyclooxygenase-2
DUF	Diabetic Foot Ulcers
DNA	Deoxyribonucleic Acid
DPLP	Data and Project Leadership
ECM	Extracellular Matrix
EGF	Epidermal Growth Factor
EGFR	Epidermal Growth Factor Receptor
eNOS	endothelial Nitric Oxide Synthase
ERK1/2	Extracellular signal-Regulated Kinase ½
FAPEMA	Maranhão Research Foundation
FGF-2	Fibroblast Growth Factor 2
FINEP	Studies and Projects Funding
GLUT-1	Glucose Transporter 1
GSH	Glutathione
H2O2	Hydrogen Peroxide
HIF-1α	Hypoxia-Inducible Factor-1 alpha
HO-1	Heme Oxygenase-1
HPX	Hydroxyproline
HUVEC	Human Umbilical Vein Endothelial Cells
IFN-γ	Interferon-gamma
IGF-1	insulin-like Growth Factor 1
IL-12	Interleukin-12
IL-1β	Interleukin-1 beta
IL-2	Interleukin-2
IL-6	Interleukin-6
IL-8	Interleukin-8
MDA	Malondialdehyde
MMP	Matrix Metalloproteinases
MMP-2	Matrix Metalloproteinase-2
MMP-9	Matrix Metalloproteinase-9
mRNA	messenger Ribonucleic Acid
MRSA	Methicillin-Resistant Staphylococcus Aureus
n.i	not informed
n.r	not reported
NF-κB	Nuclear Factor kappa B
NO	Nitric Oxide
NOS	Nitric Oxide Synthase
Nrf2	Nuclear factor erythroid 2-related factor 2
PCC	Population, Concept, Context
PDGF	Platelet-Derived Growth Factor
PI3K/Akt	Phosphoinositide 3-Kinase/Protein Kinase B
PPGCS	Graduate Program in Health Sciences
PPGST	Graduate Program in Health and Technology
PRISMA	Preferred Reporting Items for Systematic Reviews and Meta-Analyses
QCRI	Qatar Computing Research Institute
RAGE	Receptor for Advanced Glycation End Products
ROS	Reactive Oxygen Species
SOD	Superoxide Dismutase
STZ	Streptozotocin
TAC	Total Antioxidant Capacity
T-AOC	Total Antioxidant Capacity
TGF-β	Transforming Growth Factor-beta
TIMP	Tissue Inhibitor of Metalloproteinases
TIMP-2	Tissue Inhibitor of Metalloproteinases-2
TNF-α	Tumor Necrosis Factor-alpha
tTG	tissue Transglutaminase
VEGF	Vascular Endothelial Growth Factor
WBC	White Blood Cells
